# Impact of the SARS-CoV-2 (COVID-19) crisis on surgical training: global survey and a proposed framework for recovery

**DOI:** 10.1093/bjsopen/zraa051

**Published:** 2021-04-15

**Authors:** Research Education INnovation in Surgery (REINS) initiative COVID-19 group, M Yiasemidou, J Tomlinson, I Chetter, Chandra Shekhar Biyani, P Abdulhannan, A Andreou, S Badiani, R Boyapati, N Da Silva, P Dickerson, C Frezzini, A Giorga, D Glassman, J Gómez Rivas, M Ho, O P James, D Kalifatidis, W Lam, C M B Lewis, A Malik, A Mavor, J Murugesan, D Panagiotou, B Patel, D B T Robinson, R Sanchez Salas, D Sharma, J Sultan, B Van Cleynenbreugel, Z Wellbelove, A Wilson

## Abstract

**Background:**

The SARS-CoV-2 pandemic had a profound impact on surgical services, potentially having a detrimental impact on training opportunities. The aim of this global survey was to assess the impact of the COVID-19 crisis on surgical training and to develop a framework for recovery.

**Methods:**

A cross-sectional, web-based survey was conducted. This was designed by a steering committee of medical educationalists and validated by a group of trainees before dissemination.

**Results:**

A total of 608 responses were obtained from 34 countries and 15 specialties. The results demonstrated major disruption in all aspects of training. The impact was greatest for conferences (525 of 608) and hands-on courses (517 of 608), but less for inpatient care-related training (268 of 608). European trainees were significantly more likely to experience direct training disruption than trainees in Asia (odds ratio 0.15) or Australia (OR 0.10) (χ^2^ = 87.162, *P* < 0.001). Alternative training resources (webinars, 359 of 608; educational videos, 234 of 608) have emerged, although trainees expressed some dissatisfaction with them. The collective responses generated a four-pillar framework for training recovery that involved: guidance from training stakeholders with the involvement of trainees; prioritization of training, especially the roles of senior surgeons/trainers; provision of access to alternative/new teaching methods; and measures to address trainee anxiety.

**Conclusion:**

Training has been greatly affected by the COVID-19 pandemic. The introduction of new teaching methods and a focus on training after the pandemic are imperative.

## Teaser

The COVID-19 pandemic has provoked great changes in surgical services, with a severe impact on surgical training. Compensatory measures were met with mild disappointment by trainees.

Training devastated in the west

## Resumen

**Antecedentes:**

La pandemia por SARS-Cov-2 tuvo un impacto profundo en los servicios de cirugía, lo que podría causar un efecto perjudicial en las oportunidades de formación. El objetivo de esta encuesta global fue evaluar el impacto de la crisis de la COVID-19 en el entrenamiento quirúrgico y desarrollar un marco para la recuperación.

**Métodos:**

Se realizó una encuesta transversal por Internet, la cual fue diseñada por un comité directivo de educadores médicos y validada por un grupo de profesionales en periodo de formación antes de su difusión.

**Resultados:**

Se obtuvieron 608 respuestas de 34 países y 15 especialidades. Los resultados demostraron importantes interrupciones en todos los aspectos del entrenamiento. El impacto fue mayor para las conferencias (525/608) y los cursos prácticos (517/608), pero menor para la formación relacionada con la atención hospitalaria (268/608). Los profesionales en periodo de formación europeos fueron significativamente más propensos a experimentar interrupciones directas en el entrenamiento que los de Asia (odds ratio 0.15) o Australia (OR 0.10) (χ^2^ = 87.162, *P* < 0.001). Han surgido recursos de formación alternativos (seminarios web: 359/608, videos educativos: 234/608), aunque los alumnos expresaron cierta insatisfacción con ellos. Las respuestas colectivas generaron un marco de cinco pilares para la recuperación de la formación que implicaron: priorización del alumno y seguridad pública; orientación de las partes interesadas en la formación con la participación de los alumnos; priorización de la formación, especialmente de las funciones de los cirujanos senior/ profesores; facilitación de acceso a métodos y medidas de enseñanza nuevos o alternativos para abordar la ansiedad de los alumnos.

**Conclusión:**

La formación se ha visto muy afectada. Es imperativo introducir nuevos métodos de enseñanza y poner énfasis en la formación después de la pandemia.

## Introduction

In December 2019, a novel coronavirus (SARS-CoV-2) was identified in Wuhan, Hubei Province, China. This virus has the capacity to cause severe acute respiratory syndrome^1^^,^^2^ and has quickly spread around the world. As a result, a coronavirus SARS-CoV-2 pandemic was declared by the WHO on 11 March^3^. Within 6 months, nearly 6 million patients have been confirmed worldwide with around 400 000 deaths^4^.

In response to the pandemic, unprecedented measures were introduced to reduce exposure to the virus^5^. Semiurgent and elective surgery, as well as endoscopy, were discontinued in many centres after relevant recommendations from professional bodies^6–12^. Educational courses^13^^,^^14^, examinations, conferences, and training rotations were cancelled^15^.

In an attempt to minimize staff numbers and operating time, many centres restricted surgery to consultants^16^. To mitigate the increased levels of postoperative adverse outcomes, several conditions that would have been treated surgically have been managed conservatively^17–20^, further reducing operative experience. Lack of face-to-face outpatient clinics and early, safe discharge of patients also had an impact on training^15^. The impact of the COVID-19 crisis on surgical education was perhaps offset by the increased use of online resources, such as webinars, videos of operative procedures, and prerecorded lectures^15^.

The primary aim of this global survey was to provide intelligence regarding the direct and indirect impact of the pandemic on surgical education and training. The secondary aim was to explore methods that might mitigate negative effects on training, during and after the pandemic.

## Methods

The Research Education Innovation in Surgery initiative conducted a cross-sectional, web-based survey to evaluate the impact of the COVID-19 crisis on surgical training. Surgical trainees from all surgical specialties (in formally recognized training posts or otherwise) around the world were invited to complete the questionnaire. After consultation with a UK National Health Service research and development department, the authors were advised that research ethics approval was not required.

## Survey content and development

The first draft of the questionnaire was developed by a steering committee of four individuals after review of the relevant literature. The aim was to design a short questionnaire (fewer than 20 questions) that could be completed in less than 10 min. After an initial meeting, 30 questions were proposed, 14 of which were later excluded, as consensus could not be reached. Following feedback from trainees, a final questionnaire including 16 questions was agreed. Survey questions addressed five domains: demographic characteristics, direct impact on training, indirect impact on training, new learning tools introduced during the pandemic, and suggestions/thoughts about the future of training after the pandemic (*Table S1*).

## Data collection

The survey was created on Google^®^ Forms (Google, Mountain View, California, USA) (all areas besides mainland China) and SurveyMonkey^®^ (SurveyMonkey, San Mateo, California, USA) (mainland China). Responses were collected between 23 April and 15 May 2020. Surgical trainees (first-year specialty trainees up to postcompletion for training fellows) working in different surgical specialties from all over the globe were invited to complete the survey via regular Twitter posts. Several surgical and research societies engaged and retweeted to their followers. The survey was disseminated in English, Spanish, Chinese, and French.

The results of the study were extracted automatically to an Excel™ spreadsheet (Microsoft, Redmond, Washington, USA) from Google^®^ Forms. The results from SurveyMonkey^®^ were added to the same spreadsheet after being translated into English. The results were accessible to the steering group.

## Data synthesis and analysis

Data from the multiple-choice questions were analysed to produce descriptive statistics. Microsoft Excel™ software was used for data analysis. A world heat map was produced using an Excel™ add-on template (IndZara)^21^. Where responses were provided in free-text form, thematic synthesis of the results was undertaken by two of the authors. A logistic regression model with co-variable adjustment for Training grade and sex was used for comparison between continents, using the open-source graphical software JASP (http://www.jasp-stats.org).

## Results

A total of 608 participants responded, from 34 countries and 15 specialties. The majority were from the UK, Australia, and Spain (*Table 1*); eight did not specify a country (non-UK/not specified). Among respondents, 202 of 608 (62.3 per cent) were men; one-third (202) were general surgery trainees, followed by trainees in urology (163, 26.6 per cent), trauma and orthopaedics (67, 11.0 per cent), and oral and maxillofacial surgery (43, 7.1 per cent). Most respondents (410, 67.4 per cent) had been working in their specialty for 3 years or more.

**Table 1:** Demographics of respondents

	**No. of respondents** **(*n =* 608)**
**Gender**	
M	379 (62.3)
F	227 (37.3)
Other	2 (0.3)
**Country**	
UK	337 (55.4)
Australia	48 (7.9)
Spain	39 (6.4)
China	29 (4.8)
India	28 (4.6)
Belgium	24 (3.9)
Hong Kong	24 (3.9)
Greece	14 (2.3)
Other	65 (10.7)
**Specialty**	
General surgery	202 (33.2)
Urology	163 (26.8)
Trauma and orthopaedics	67(11.0)
Oral maxillofacial surgery	43 (7.1)
Vascular surgery	29 (4.8)
Obstetrics and gynaecology	22 (3.6)
Ear nose and throat surgery	18 (3.0)
Plastic surgery	13 (2.1)
Other	51 (8.4)
**Surgical experience (years)**	
< 3	198 (32.6)
≥ 3	410 (67.4)

Values in parentheses are percentages.

### Disruption of training: direct effect

**Fig. 1:**
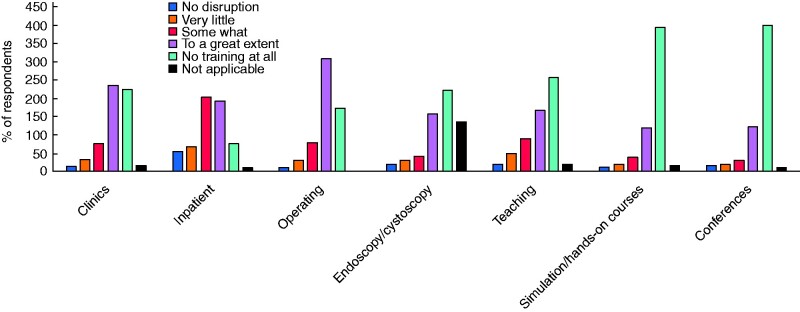
Impact of COVID-19 pandemic on aspects of surgical training

The majority of respondents reported significant disruption or complete discontinuation of all aspects of surgical training (*Fig. 1*). Particularly affected were attendance at conferences (525 of 608, 86.3 per cent) and hands-on courses such as simulation (517 of 608, 85.0 per cent). Outpatient clinic training (462 of 608, 76.0 per cent), operative experience (483 of 608, 79.4 per cent), endoscopy/cystoscopy (379 of 608, 62.3 per cent), regional teaching (428 of 608, 70.4 per cent), and training relating to inpatient care (268 of 608, 44.1 per cent) were also greatly affected.

**Fig. 2:**
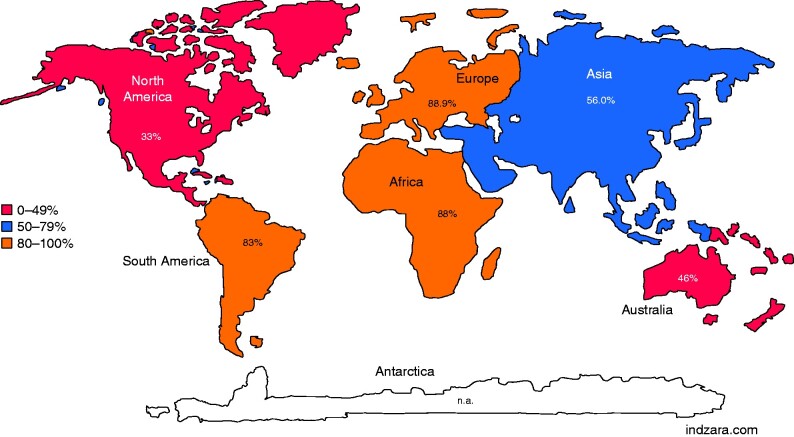
Percentage of respondents reporting a high impact of COVID-19 pandemic on operating room training by continent n.a., Not available.

Training in the operating theatre appeared to be less compromised in Australasia. Where the response was ‘affected to a great extent’ or ‘no training at all’, 22 of 48 trainees (46 per cent) said this was the case in Australia and 56 of 100 (56.0 per cent) in Asia, compared with 384 of 432 (88.9 per cent) in Europe (*Fig. 2*). As North America (6 respondents), South America (6), and Africa (8) were under-represented, no accurate conclusion could be reached for these continents. European trainees were significantly more likely to experience direct training disruption due to COVID-19 than trainees in Asia (odds ratio 0.15) or Australia (OR 0.10) (χ^2^ = 87.162, *P* < 0.001).

### Disruption of training: indirect effect

**Fig. 3:**
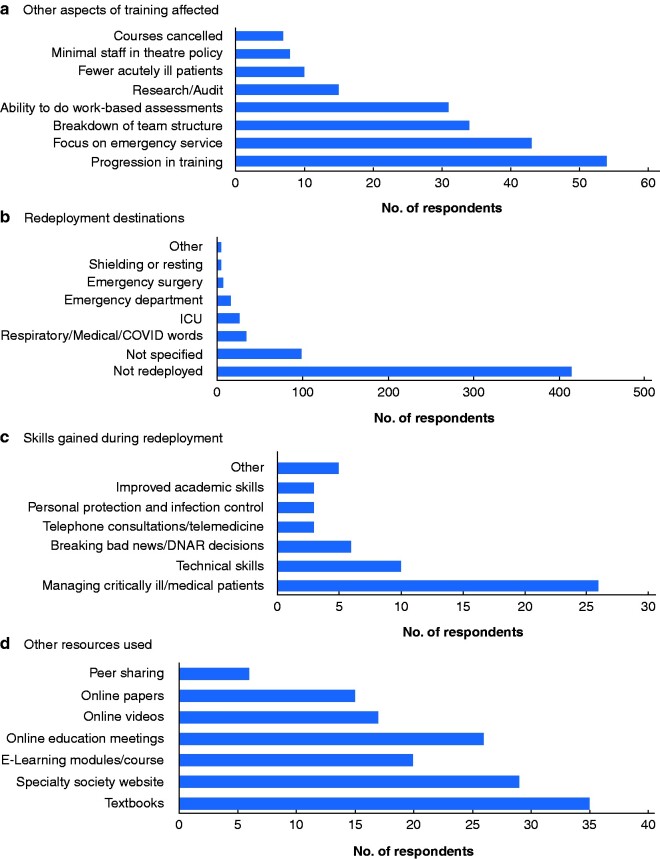
Indirect effect of COVID-19 pandemic training **a** Other aspects of training affected, **b** redeployment destinations, **c** skills gained during redeployment, and **d** other resources used for teaching. DNAR, do not attempt resuscitation.

The most common indirect consequence reported by trainees (54 of 202, 26.7 per cent) was interruption to career progression (*Fig. 3a*). Cited reasons included: discontinuation of rotation, examination cancellation, and modification of the recruitment process (no face-to-face interviews owing to social distancing rules). Trainees undertaking higher degrees had to return to clinical duties, halting their academic progress. Providing emergency care related to COVID-19 deprived trainees of the few ongoing elective activities (43 of 202, 21.3 per cent), and redeployment to other specialties or change of work duties within the same specialties disrupted clinical team coherence leading to fewer opportunities for mentoring by senior surgeons (34 of 202, 16.8 per cent) or case-based discussions (31 of 202, 15.3 per cent).

Research and audit activities were reported as no longer a priority by a small number of trainees (15 of 202, 7.4 per cent) (*Fig. 3a*). A reduction in emergency admissions reduced exposure and training opportunities in acute surgical management (10 of 202, 5.0 per cent). Several centres applied a policy of minimal staff in theatre, in an attempt to minimize the exposure of healthcare staff to aerosol-producing procedures, such as intubation and pneumoperitoneum. This restricted theatre access for trainees, further limiting training opportunities (8 of 202, 4.0 per cent).

Redeployment was reported by 193 of 608 respondents (31.7 per cent) (*Fig. 3b*). The most common destinations of redeployment were the medical/respiratory wards (treating patients with COVID-19) (34 of 193, 17.6 per cent), intensive care (26 of 193, 13.5 per cent), and the emergency department (16 of 193, 8.3 per cent). Trainees acquired new skills during redeployment, including management of critically ill patients (COVID-19), and technical skills such as central venous and arterial line insertions.

### Factors affecting provision of training during pandemic

**Fig. 4:**
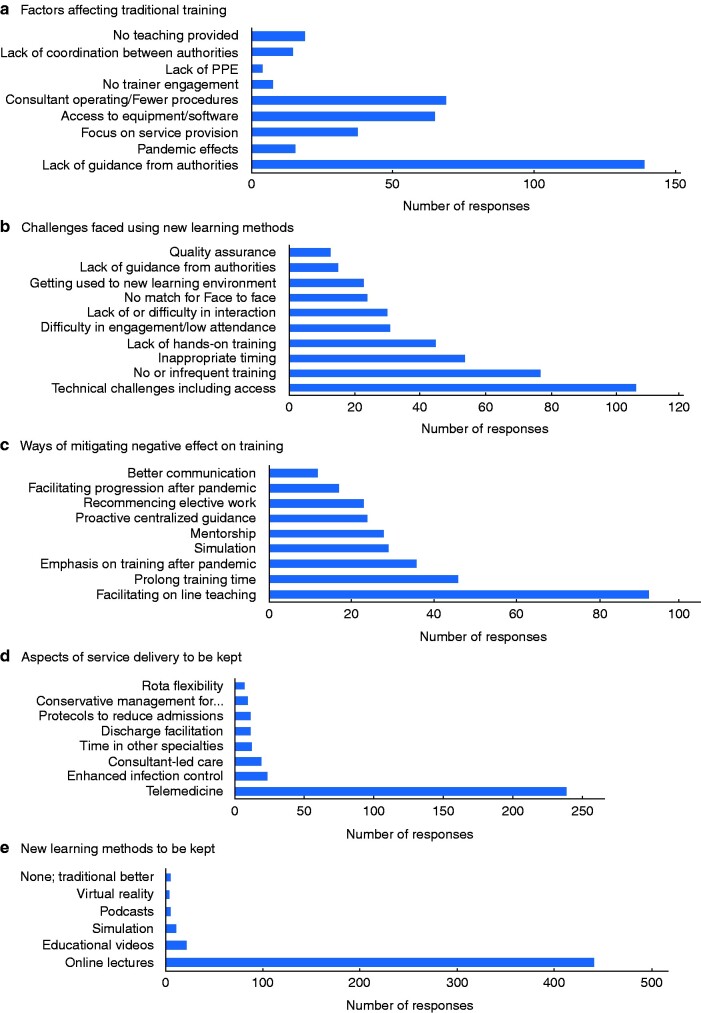
Factors affecting provision of training and future of surgical training **a** Factors affecting traditional training, **b** challenges faced using new learning methods, **c** ways of mitigating negative effect on training, and **d** aspects of service delivery and **e** new learning methods to be kept in place after the pandemic. PPE, personal protective equipment.

Lack of guidance from local or national training authorities was identified as having a negative impact on training during the pandemic by 139 of 373 respondents (37.3 per cent) (*Fig. 4a*). The reduced elective and emergency caseload and a consultant-only operating policy led to fewer operative training opportunities (69 of 373, 18.5 per cent). Limited access to appropriate equipment was also an issue, including lack of IT equipment, technical troubleshooting with internet connections, and lack of simulators (65 of 373, 17.4 per cent). Other factors included the focus on service provision (38 of 373, 10.2 per cent), and discontinuation of teaching and training because of social distancing (19 of 373, 5.1 per cent). A small number of respondents (15 of 373, 4.0 per cent) felt that there was lack of communication and coordination between training and other authorities; this often resulted in duplication (such as online sessions on the same topic) or contradictory information being passed on to trainees. Some trainees expressed the opinion that discontinuation of training was sensible and inevitable owing to the effects of the pandemic (16 of 373, 4.3 per cent).

**Fig. 5:**
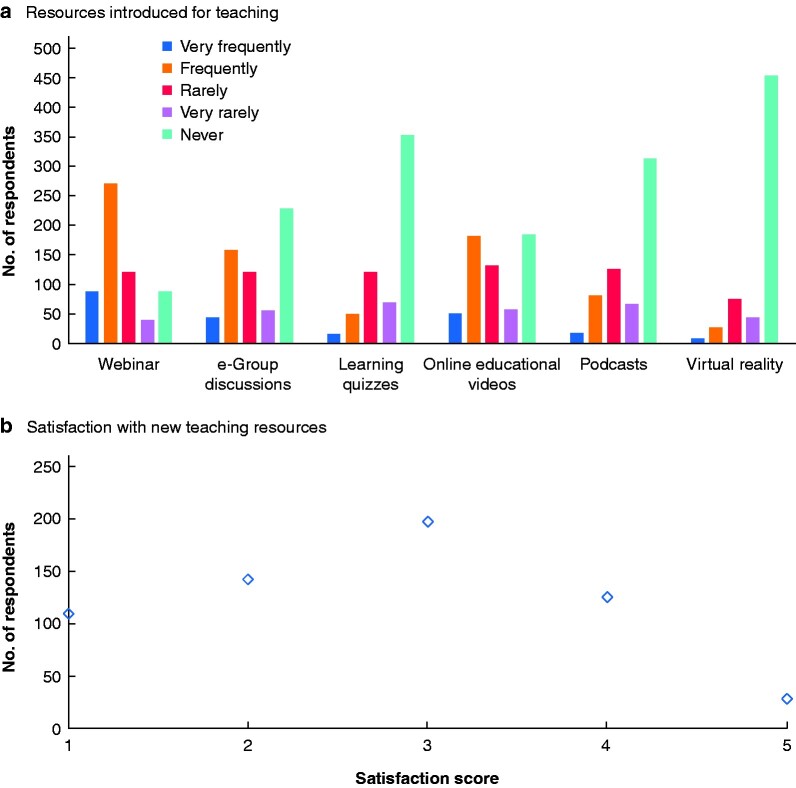
New teaching resources used during COVID-19 pandemic **a** Resources introduced for teaching and **b** satisfaction with new teaching resources (1, not satisfied at all; 5, very satisfied).

Surgical trainees adjusted rapidly, identifying new educational opportunities including: webinars (359 of 608, 59.0 per cent), online educational videos (234 of 608, 38.5 per cent), virtual reality resources (34 of 608, 5.6 per cent), and online learning quizzes (64 of 608, 10.5 per cent) (*Fig. 5a*). Other less frequently accessed educational resources included: textbooks, e-books, and e-libraries (35 of 148, 23.6 per cent); updates/guidelines on surgical and other societies’ websites (29 of 148, 19.6 per cent); small group, interactive, online teaching sessions (26 of 148, 17.6 per cent); prerecorded teaching sessions (17 of 148, 11.5 per cent); online papers/journals (15 of 148, 10.1 per cent); and peer sharing, such as online forums and WhatsApp^®^ (WhatsApp, Menlo Park, California, USA) groups (6 of 148, 4.1 per cent) (*Fig. 3d*).

The introduction of novel training methods (or enhancement of pre-existing training methods such as simulation) was not without challenges, including technical issues in accessing online educational materials (106 of 418, 25.4 per cent), small numbers of virtual or simulation training sessions (77 of 418, 18.4 per cent), inappropriate timing of webinars (54 of 418, 12.9 per cent), lack/inability to receive hands-on training on simulated patients or simulators (45 of 418, 10.8 per cent), difficulty in engaging in and maintaining concentration during online sessions (31 of 418, 7.4 per cent), and lack of interaction during online sessions (30 of 418, 7.2 per cent) (*Fig. 4b*). These may have led to the majority of respondents being dissatisfied with the alternative (to standard clinical training) educational resources (254 of 608, 41.8 per cent) (*Fig. 5b*).

### Future of surgical training

Respondents felt that factors which may help mitigate the negative effects of the pandemic on training included: ongoing online teaching sessions (92 of 307, 30.0 per cent), prolonging training time (46 of 307, 15.0 per cent), prioritizing training and educational activities over service provision after the pandemic (36 of 307, 11.7 per cent), increased use of simulation (29 of 307, 9.4 per cent), mentorship by senior surgeons (28 of 307, 9.1 per cent), proactive guidance from training authorities (24 of 307, 7.8 per cent), and recommencing elective work (23 of 307, 7.5 per cent) (*Fig. 4c*).

New educational resources, such as online lectures and educational videos, were popular (441 of 489, 90.1 per cent) (*Fig. 4e*). With regard to service delivery, a significant number of respondents (239 of 332, 72.0 per cent) felt that telemedicine (virtual clinics, virtual multidisciplinary team (MDT) meetings) should continue (*Fig. 4d*). A small number supported the continuation of enhanced infection control (23 of 332, 6.9 per cent) and consultant-led care (19 of 332, 5.7 per cent) after the pandemic.

From the free-text comments, there appeared to be understanding that the negative impact of the COVID-19 pandemic on training was inevitable, primarily to maintain trainee safety. There was, however, frustration about the perceived lack of response by training authorities in addressing this. UK trainees in particular were frustrated by the cancellation of surgical rotations and modification of the recruitment process to higher surgical training. Many respondents highlighted the need for training authorities and trainers to emphasize training, and to have a structured plan to prioritize training during the post-COVID-19 period so that trainees would be assisted to make up for experience not gained during the pandemic. Mentorship by senior doctors, use of simulation, e-learning methods, and telemedicine were once again mentioned and favoured.

## Discussion

This survey assessed the global impact of the COVID-19 pandemic on surgical training. It included responses from 34 countries and 15 different surgical specialties. Although there was variation between countries, it provided evidence of widespread global disruption of all aspects of surgical training. Similar results have been reported in national or specialty studies^15^^,^^22–30^. Alternative resources have developed rapidly; interestingly, trainees expressed some dissatisfaction with these for a variety of reasons.

Experience from previous pandemics has shown that disruption to training may be prolonged^31^, so the development of a strategy for recovery of training after the pandemic is important. Based on the present survey, it is suggested that this recovery should include the following elements.

This survey identified a lack of guidance from organizations and individuals responsible for training as one of the main obstacles to training during the pandemic. Although there was sufficient guidance in regards to service provision^17^, directives for training were slow to emerge. Several societies launched online educational platforms^32^, often in an uncoordinated way resulting in duplication.

Training stakeholders need to improve communication and coordinate activities, producing widely accepted guidelines with the participation of trainees. Timely communication and exchange of complete, accurate information between learners, hospital management, educators, and training committees are vital. Standards to address training needs during a pandemic have been proposed, to include prioritization of healthcare system welfare, promotion of learner welfare, maximization of educational value, and transparent communication^33^.

Hospitals worldwide should be encouraged to emphasize the importance of training alongside service provision once the pandemic is over. The hiatus of elective surgery during the COVID-19 crisis has created a significant backlog of patients. Under these circumstances surgeons may be apprehensive in providing training in the operating theatre owing to time restraints and service provision commitments. Trainers should exhibit strong leadership^34^, and be actively encouraged to train and mentor young surgeons both in and outside the operating theatre. Interventions to improve the efficiency of service provision should be developed. Virtual clinics, consultations, wider use of MDTs, and telemedicine^35^ might all contribute in this way. Mentorship was mentioned repeatedly in the survey, and seems crucial in re-establishing effective and efficient surgical training.

Webinars, educational videos, e-libraries, and simulation are popular among trainees^23^^,^^35^, and their use should be facilitated during and after the pandemic^32^. Hospitals should commit to providing trainees with access to a high-quality internet network, up-to-date hardware and software, and readily available simulators. Simulation centres should consider expanding their working hours or find alternative methods to give trainees access in evenings and at weekends. Lack of out-of-hours access has been identified previously as one of the barriers to simulator use by trainees^36^^,^^37^. In addition, simulation training has been shown to be more effective in the presence of a trainer (instead of self-driven). Appointing trainers for simulation sessions may accelerate training recovery after the pandemic^37^^,^^38^.

Respondents suggested that restarting elective activities should be undertaken cautiously while maintaining patient and staff safety as a priority. Adequate personal protection equipment and avoidance of face-to-face interaction when possible seem imperative, as long as large numbers of patients with COVID-19 are still being reported. The association between the overall success of the response to a pandemic (small number of patients, preservation of public safety) and surgical training was apparent in the responses and comparisons between Europe and Australia/Asia. The significantly lesser impact on training in Australia and Asia compared with Europe may well reflect differences in numbers of affected patients between continents during the survey period^39^.

In addition to the anxiety caused by the pandemic itself^40^, surgical trainees experienced worry about career progression owing to cancellation of examinations^41^ and training rotations^42^. Annual review and recruitment processes had to be modified at very short notice to comply with governmental public health measure regulations^42^. Consideration must be given to assessment of competency for progression. Prolongation of training (popular among responders) might then be offered as a voluntary option to trainees. Hospitals should offer well-being sessions (such as ‘stop stations’^43^) for staff to help mitigate the adverse effects of pandemics on their mental health.

**Fig. 6:**
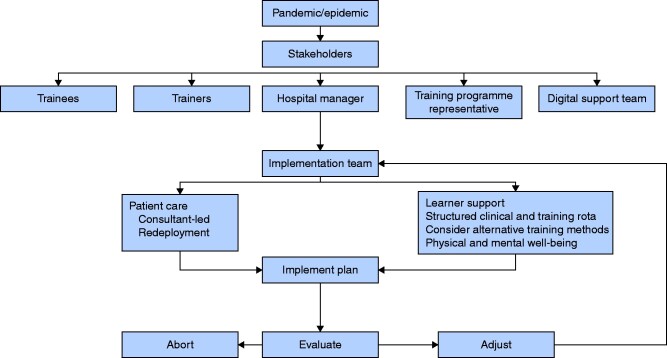
Recovery plan flow chart

The recent COVID-19 outbreak demonstrated the vulnerability of many healthcare systems in managing education and patient care when a crisis occurs. Politicians and healthcare leaders used reactive policies to deal with rapidly developing situations that stemmed from lack of preparation. Taking a proactive approach rather than having a reactive attitude may minimize unintended effects such as the curtailment of surgical training. *Fig. 6* illustrates a proposal for policy development. Having an approach that involves all stakeholders should pay added dividends at the time of adversity. Effective educational strategies to address trainees’ needs while protecting patients are required.

This study has limitations. Some parts of the world were under-represented (Africa and America) and, although efforts were made to ensure that the study was available in several languages, it is acknowledged that language barriers may have limited participation. As this was an online survey, parts of the world with limited access to the internet or computer equipment could not participate. The low response rates from North America may have been due to lack of retweets from groups based in North American countries. A shortage of collaborators/regional leads from North America and some European countries may have been a further contributing factor. The volume of data, however, still seems adequate to indicate the COVID-19 pandemic resulted in global disruption of surgical training and has facilitated the provision of realistic suggestions on how the impact of this disruption can be mitigated in future.

## Collaborators

Steering committee: M. Yiasemidou (Leeds Teaching Hospitals, Leeds, UK; University of Hull, Hull, UK; Hull University Teaching Hospitals NHS Trust, Hull, UK); J. Tomlinson (Sheffield Teaching Hospitals, Sheffield, UK); I. Chetter (University of Hull, Hull, UK; Hull University Teaching Hospitals NHS Trust, Hull, UK); C. Shenkar Biyani (Leeds Teaching Hospitals, Leeds, UK). Regional leads and collaborators: P. Abdulhannan (Leeds Teaching Hospitals, Leeds, UK); A. Andreou (York Teaching Hospitals NHS Trust, York, UK); S. Badiani (Bankstown‐Lidcombe Hospital, Sydney, New South Wales, Australia); R. Boyapati (Royal Surrey County Hospital, Guildford, UK); N. Da Silva (Leeds Teaching Hospitals, Leeds, UK); P. Dickerson (York Teaching Hospitals NHS Trust, York, UK); C. Frezzini (Health Education East Midlands Deanery, Leicester, UK); A. Giorga (Leeds Teaching Hospitals, Leeds, UK); D. Glassman (Sheffield Teaching Hospitals, Sheffield, UK); J. Gómez Rivas (La Paz University Hospital, Madrid, Spain); M. Ho (Leeds Teaching Hospitals, Leeds, UK); O. P. James (Royal Gwent Hospital, Newport, UK); D. Kalifatidis (Asklepieio Voulas, Athens, Greece); W. Lam (Queen Mary Hospital, Li Ka Shing Faculty of Medicine, University of Hong Kong, Hong Kong, China); C. M. B. Lewis (Leeds Teaching Hospitals, Leeds, UK); A. Malik (Mid Yorkshire NHS Trust, Wakefield, UK); A. Mavor (Leeds Teaching Hospitals, Leeds, UK); J. Murugesan (Leeds Teaching Hospitals, Leeds, UK); D. Panagiotou (Aristotle University of Thessaloniki, Thessaloniki, Greece); B. Patel (Barts Cancer Institute, Queen Mary University, University of London, UK); D. B. T. Robinson (Wales Deanery PGMDE School of Surgery, Health Education and Improvement Wales, Cardiff, UK; Swansea University Medical School, Sketty, UK); R. Sanchez Salas (L’Institut Mutualiste Montsouris, Paris, France); D. Sharma (Government Medical College and Allied Hospitals, Jabalpur, India); J. Sultan (Hull University Teaching Hospitals NHS Trust, Hull, UK); B. Van Cleynenbreugel (University Hospitals Leuven, Leuven, Belgium); Z. Wellbelove (Hull University Teaching Hospitals NHS Trust, Hull, UK); A. Wilson (City Hospitals, Sunderland, UK).

*Disclosure.* The authors declare no conflict of interest.

## Supplementary material

Supplementary material is available at *BJS Open* online.

## Supplementary Material

zraa051_Supplementary_Data

## Notes

Members of the Research Education INnovation in Surgery (REINS) initiative COVID-19 group are co-authors of this study and are listed under the heading Collaborators
