# Supplementary material for: Impact of the SARS-CoV-2 (COVID-19) crisis on surgical training: global survey and a proposed framework for recovery
Source: BJS Open. 2021 Apr 15;5(2):zraa051. doi: 10.1093/bjsopen/zraa051 (PMC8047098; doi:10.1093/bjsopen/zraa051)
Supplement: zraa051_Supplementary_Data [file zraa051_supplementary_data.pdf]

Surgical training during COVID

The COVID 19 outbreak had a significant impact on surgical training. This survey aims to quantify this and help find ways to overcome this. Thank you for taking the time to complete this. By doing so you give consent for the anonymised data to be analysed and disseminated (including academic and other publications).

* Required

1. What is your specialty? *

____

2. What is your grade? *

____

3. What is your gender *

Mark only one oval.

- ( ) Female
- ( ) Male
- ( ) Other: _______________________________

4. Which region (deanery if in the UK, country if non UK) are you based in? *

____

5. How has the COVID-19 crisis disrupted the provision of training? *

Mark only one oval per row.

	No disruption	Very little	Somewhat	To a great extent	No training at all	Not applicabl
Out-patient clinics	⬭	⬭	⬭	⬭	⬭	⬭
In-patient (ward round, case-based discussion)	⬭	⬭	⬭	⬭	⬭	⬭
Operating (surgical training in theatre)	⬭	⬭	⬭	⬭	⬭	⬭
Endoscopy/cystoscopy/ endovascular etc. training	⬭	⬭	⬭	⬭	⬭	⬭
Regional Teaching sessions	⬭	⬭	⬭	⬭	⬭	⬭
Simulation/hands on courses	⬭	⬭	⬭	⬭	⬭	⬭
Conferences	⬭	⬭	⬭	⬭	⬭	⬭

6. Please mention other aspects of training that may have been disrupted and how

7. Have you been redeployed during this time and if yes have you learned any new skills? *

8. What resources have been introduced to deliver training in light of reduced face-to-face teaching? *

Mark only one oval per row.

	Very frequently	Frequently	Rarely	Very rarely	Never
Webinar					
e-group discussion					
Interactive learning quizzes					
On line educational videos					
Podcasts					
Virtual reality					

9. Any other resources you may have used?

10. Are there any aspects of the service delivery introduced due to the COVID-19 crisis that would be useful after the crisis? *

11. What new learning method experienced during this crisis would you like to incorporate in the regular training session? *

12. How satisfied are you with the new methods of training delivery? *

Mark only one oval.

	1	2	3	4	5	
Not at all satisfied	⬭	⬭	⬭	⬭	⬭	Very satisfied

13. What challenges did you face with the new method of training delivery? *

14. If any, what factors affected provision of training? e.g. lack of guidance from local training authority, lack of access to appropriate computer equipment etc. *

15. Do you have any other ideas on how the negative impact on training can be mitigated? *

16. Any other ideas, thoughts or questions?

This content is neither created nor endorsed by Google.

Google Forms
